# Organic fertilizer altering root trait and microbial composition to promote the compactness tolerance of peanut

**DOI:** 10.3389/fpls.2026.1783977

**Published:** 2026-04-22

**Authors:** Haiyan Liang, Yinglong Chen, Liyu Yang, Qi Wu, Miao Liu, Jialei Zhang, Pu Shen

**Affiliations:** 1 Shandong Peanut Research Institute/State Key Laboratory of Nutrient Use and Management, Shandong Academy of Agricultural Sciences, Qingdao, China; 2 School of Agriculture and Environment, and UWA Institute of Agriculture, The University of Western Australia, Perth, WA, Australia; 3 Institute of Crop Germplasm Resources, Shandong Academy of Agricultural Sciences, Jinan, China

**Keywords:** *Arachis hypogaea*, compaction, organic fertilizer, root morphology, soil microbial diversity, soil property

## Abstract

Continuous cultivation and excessive chemical fertilizer use have led to increased soil compaction in peanut (*Arachis hypogaea* L.) fields, significantly hindering plant growth and development. Organic fertilizer can improve soil nutrient content, aeration, and overall soil environments, thereby promoting healthy plant growth. However, the mechanisms underlying the effects of substituting chemical fertilizer with organic fertilizer on peanut growth under compaction stress are still elusive. To investigate the responses of peanut root traits, soil physicochemical properties, and microbial community structure to organic fertilizer substitution under soil compaction, a pot experiment was conducted using two compaction levels (1.2 and 1.6 g/cm^3^) and three organic fertilizer rates (0, 120, 240 g/pot) in Laixi, Shandong Province, a major peanut producing area. Results showed that organic fertilizer significantly improved plant biomass, height, stem diameter, and nodule fresh weight during the flowering and podding stages. Root traits including total root length, total surface area, and root volume, were significantly increased, especially at the podding stage (p < 0.05). The number of xylem vessels increased under 1.2 g/cm^3^ compaction but showed no significant change under 1.6 g/cm^3^. Soil available phosphorus and potassium contents increased with higher fertilizer rates. Distance-based redundancy analysis (RDA) showed that rhizosphere bacterial and fungal communities were affected by soil physical and chemical properties. Partial least squares path modeling (PLS-PM) indicated that soil organic matter was positively correlated with soil enzyme activity, which indirectly enhanced fungal diversity, while bacterial diversity showed negative correlation with soil organic matter. In conclusion, applying organic fertilizer in accordance with soil compaction levels offers an efficient strategy to improve soil microbial structure, enhance soil fertility, and promote peanut growth under compacted field conditions.

## 1 Introduction

Peanut (*Arachis hypogaea* L.) is an important global oil and economic crop. Major peanut production areas are typically characterized by large-scale cultivation and mechanized farming. However, with the continuous reduction in arable land, practices such as frequent cultivation and excessive fertilization have accelerated soil degradation processes, leading to environmental issues such as soil acidification and compaction (Nadeem Shah et al., 2023). Among these issues, the loss of soil organic matter further deteriorates soil physical properties, resulting in decreased soil stability (Zhao et al., 2025). Soil compaction stress, a primary factor closely linked to the decline in soil stability, exacerbates the degradation of soil physical structure (Zhang et al., 2024). Changes caused by compaction stress—such as reduced soil particle stability, decreased availability of moisture and nutrients, and impaired aeration, not only limit normal plant growth and yield but may also trigger further degradation processes including soil erosion, organic matter loss, and increased soil strength. Many studies have shown that organic carbon plays a vital role in preventing soil compaction and enhancing soil permeability and resistance (Zhang et al., 2024; Zhao et al., 2025). Zhang et al. (2024) and others found that organic carbon helps mitigate the negative effects of mechanical operations on soil physical properties by increasing soil elasticity. Additionally, the distribution of soil organic matter (SOM) among different components, such as particulate and mineral-associated organic matter, can affect soil permeability and structural stability (Startsev et al., 2020). The use of organic fertilizer to replace chemical fertilizer can effectively alleviate these issues (Nadeem Shah et al., 2023). However, the effects of organic fertilizer substitution under compaction stress in peanut fields remain unclear.

Peanut is a vital oil and economic crop in China, with an annual output exceeding 18.05 million tons since 2020, accounting for 33% of the global total (Figueiredo et al., 2017; Zhao et al., 2025). Peanut is also an important industrial raw material in China and play a significant role in the country’s import and export trade of oil crops. However, continuous cultivation and unreasonable fertilization in peanut fields have led to increasing soil compaction, which significantly affects peanut growth and development. Studies have shown that soil compaction alters the original soil physical structure by reducing porosity, rearranging aggregates, impairing aeration, increasing permeability resistance, and intensifying anaerobic conditions (Shen et al., 2020; Colombi and Walter, 2016). Changes in soil physical properties affect root structure, thereby impacting root growth and the absorption and utilization of water and nutrients (Xiong et al., 2020). Research has found that increased soil compaction inhibits field crop root growth and alters root phenotypes by reducing the number of axial and lateral roots, impeding lateral root development, thickening the main root diameter, and decreasing root vitality and nutrient absorption capacity (Colombi et al., 2017; Xiong et al., 2020). Alterations in root apex geometry serve as an adaptive strategy to penetrate compacted soil layers. These anatomical changes include cortical cell deformation, invaginations, and alterations in the cross-sectional areas of both the cortex and vascular cylinder. These changes also limit potential infection sites for rhizobia, altering the composition and function of rhizosphere microorganisms. This leads to a significant reduction in peanut root nodules, decreased nitrogenase activity, and diminished nitrogen-fixing capacity of nodules (Siczek and Lipiec, 2011; Torabian et al., 2019). The application of organic fertilizer can improve soil nutrients, aeration, and other environmental conditions, promoting soil quality improvement, enhancing microbial metabolic function and diversity, and improving crop root nutrient absorption to promote plant growth. However, the mechanisms by which organic fertilizer replacing chemical fertilizer affects peanut plants, soil, and microorganisms in compacted peanut fields remain unclear. Therefore, to explore the response mechanisms of peanut root traits, soil physical and chemical properties, and bacterial community structure to organic fertilizer replacement under soil compaction stress, a replacement experiment with varying compaction levels was conducted in Laixi. The main objectives were: (1) to determine whether organic fertilizer application can promote peanut root development, nutrient absorption, and stress resistance; and (2) to assess whether organic fertilizer application can enhance stress resistance by altering soil microbial community structure and function, as well as soil enzyme activity.

## 2 Materials and methods

### 2.1 Experimental site and design

This study was conducted in a greenhouse at the Laixi Experimental Station of the Shandong Peanut Research Institute, China (36°48’47’’N, 120°30’17’’E) in June 2023 to investigate the effect of organic fertilizer on peanut roots and the rhizosphere microbiome in compacted soil. The mean annual temperature in this region is 11.7°C, with an annual precipitation of 635.8 mm. Soil samples were collected from a depth of 0–20 cm in a peanut agricultural field. The soil texture in the topsoil layer is silt loam (46.17% sand, 47.57% silt, and 6.26% clay), with pH 7.04 (1:2.5 soil: water suspension), organic C 6.09 g/kg, available N 16.74 mg/kg, available P 20.86 mg/kg, and available K 58.26 mg/kg.

The peanut variety Huayu 33 was selected for the experiment. A potted planting method was used, with plastic buckets (top diameter 37 cm, bottom diameter 28 cm, height 34 cm) drilled with holes at the bottom for ventilation and drainage. Four seeds were planted in each pot, and three seedlings exhibiting consistent growth were selected after emergence. The experiment included two soil compaction levels and three organic fertilizer treatments. The two soil compaction levels were based on the local peanut field conditions and previous study results: a bulk density of 1.2 g/cm^3^ was considered normal soil compaction, and 1.6 g/cm^3^ was considered soil compaction stress. Each soil compaction treatment included three organic fertilizer application levels, applied before sowing. All treatments were arranged in a randomized block design with three replicates. Detailed information and abbreviations for each treatment are listed in **Table 1**.

**Table 1:** experimental design of the study.

Soil compaction level	Organic fertilization
Normal soil compaction (1.2 g/cm^3^)	0 g/pot, CK
	Organic feralization with 120 g/pot, T1
	Organic feralization with 240 g/pot, T2
Soil compaction stress (1.6 g/cm^3^)	0 g/pot, CK1
	Organic feralization with 120 g/pot, T3
	Organic feralization with 240 g/pot, T4

### 2.2 Plant sample and measurements

Plants were sampled at the flowering stage (52 days after sowing, DAS) and the podding stage (74 DAS). Three plants from each pot were sampled and dried in an oven at 75 °C for 3 days to measure dry aboveground biomass (Liang et al., 2024). Aboveground biomass was weighed and digested in a mixture of concentrated H_2_SO_4_ and H_2_O_2_. Nitrogen content was determined by the Kjeldahl method.

At each plant sampling (52 and 74 DAS), root samples were also collected to assess root morphology and root ultrastructure. Roots in each sample were gently washed with deionized water to remove soil, then divided into two parts. One part of the whole root system was scanned with an Epson scanner (modified Optical Scanner STD 4800, Epson, Matsumoto, Japan) and analyzed using WinRhizo software (Regent Instruments Inc., Qúebec, Canada) to obtain total root length, total root volume, and total root surface area. For the other part, ultrastructural micrographs of root tissues were prepared following the method of Zhang et al. (2015). Small root tissues were fixed in 2.5% (v/v) glutaraldehyde solution at 4 °C overnight. Samples were then washed three times with phosphate-buffered saline (PBS) at pH 6.8, followed by fixation with 2% (m/v) osmium tetroxide solution for 4 hours under a fume hood. After rinsing with PBS, samples were dehydrated through a graded ethanol series. Finally, the specimens were embedded in paraffin wax and polymerized at 60 °C. Ultra-thin sections were sliced using a microtome (Leica, RM 2016, Germany) and double-stained with 1.0% uranyl acetate followed by 5.0% (w/v) lead citrate. Root tissues were observed with a transmission electron microscope (Olympus, BX53, Japan).

### 2.3 Soil sampling and measurements

At each plant sampling stage, soil samples were collected by gently shaking roots from each pot to measure soil properties and enzyme activity. Rhizosphere soil, defined as the soil attached to the root surface, was carefully collected by brushing it off the roots, removing all visible plant and root material, and stored at - 80°C for microbial molecular analysis (Zhang et al., 2023).

Soil pH was measured with a pH electrode (PB-10, Sartorius, Göttingen, Germany). Soil available N (AN) available P (AP) and available K (AK) were analyzed using the method of Bao (2000). Soil CEC was determined using the sodium acetate (NaOAc) method (Chapman, 1965). Soil enzyme activities, including urease (UE), catalase (CAT), and β-glucosidase (β-GC), were measured using commercial kits (Hou et al., 2020).

### 2.4 DNA extraction, amplification, and high-throughput sequencing of bacterial 16S rRNA and fungal ITS

Total genomic DNA was extracted from soil samples using the Soil DNA Kit (Tiangen Biotech, Beijing, China) according to the manufacturer’s instructions. The purity and concentration of the DNA were assessed using a Nanodrop One (Thermo Fisher Scientific, MA, USA). The hypervariable V3–V4 region of the bacterial 16S rRNA gene was amplified with primer pairs 343F (5’-TACGGRAGGCAGCAG-3’) and 798R (5’-AGGGTATCTAATCCT-3’) (Li et al., 2021). To amplify the fungal ITS2 region, primer pairs ITS1F (5’-CTTGGTCATTTAGAGGAAGTAA-3’) and ITS4R (5’-TCCTCCGCTTATTGATATGC-3’) were used. The purified polymerase chain reaction (PCR) amplicons were then sequenced on the Illumina platform by Guangdong Magigene Biotechnology Co., Ltd. (Guangzhou, China).

The PCR thermocycling program was as follows: 5 min at 94 °C, followed by 30 cycles of 30 s at 94 °C, 30 s at 52 °C, and 30 s at 72 °C, with a final extension at 72 °C for 7 min and a hold temperature of 4 °C. PCR products from each sample were examined by electrophoresis on a 1.5% agarose gel to assess fragment length and concentration. The PCR mixture was then purified using the E.Z.N.A.^®^ Gel Extraction Kit (Omega, USA), with target DNA fragments eluted in TE buffer. Sequencing raw data obtained in this study were submitted to the National Center for Biotechnology Information Sequence Read Archive (registration number: PRJNA1353603).

### 2.5 Data treatment and analysis

Raw bacterial and fungal sequence reads were processed and analyzed using fastp (an ultra-fast all-in-one FASTQ preprocessor, version 0.14.1, https://github.com/OpenGene/fastp) to perform sliding window quality trimming (W4 -M20) separately on raw reads from both ends of paired-end data. Concurrently, primers were removed from the data using cutadapt software (https://github.com/marcelm/cutadapt/) based on primer information at the 5’ and 3’ ends of sequences, thus obtaining quality-controlled paired-end clean reads. Low-quality sequences with an average quality score below 20 and reads shorter than 50 bp were excluded. The remaining sequences were clustered into Operational Taxonomic Units (OTUs) with a cutoff of 97% similarity. Representative OTU sequences were identified using the SILVA database (http://www.arb-silva.de) with the RDP Classifier algorithm for bacteria and the UNITE database for fungi, applying a confidence threshold of 0.8. Singleton OTUs containing only one sequence across all samples were removed prior to subsequent analyses. Coverage, richness (Chao1 index), and diversity (Shannon index) were used to estimate the alpha diversity of each sample. Using the OTU abundance table, Principal Coordinates Analysis (PCoA) and PERMANOVA based on Bray-Curtis dissimilarity were performed with the vegan and ggplot2 packages in R (v.3.6.3). A Mantel test was conducted to assess relationships between soil physicochemical properties and microbial communities based on the OTU abundance table using the vegan package in R.

## 3 Results

### 3.1 The effect of organic fertilizers under soil compaction on peanut underground and root growth, biomass and N uptake

**Figure 1:**
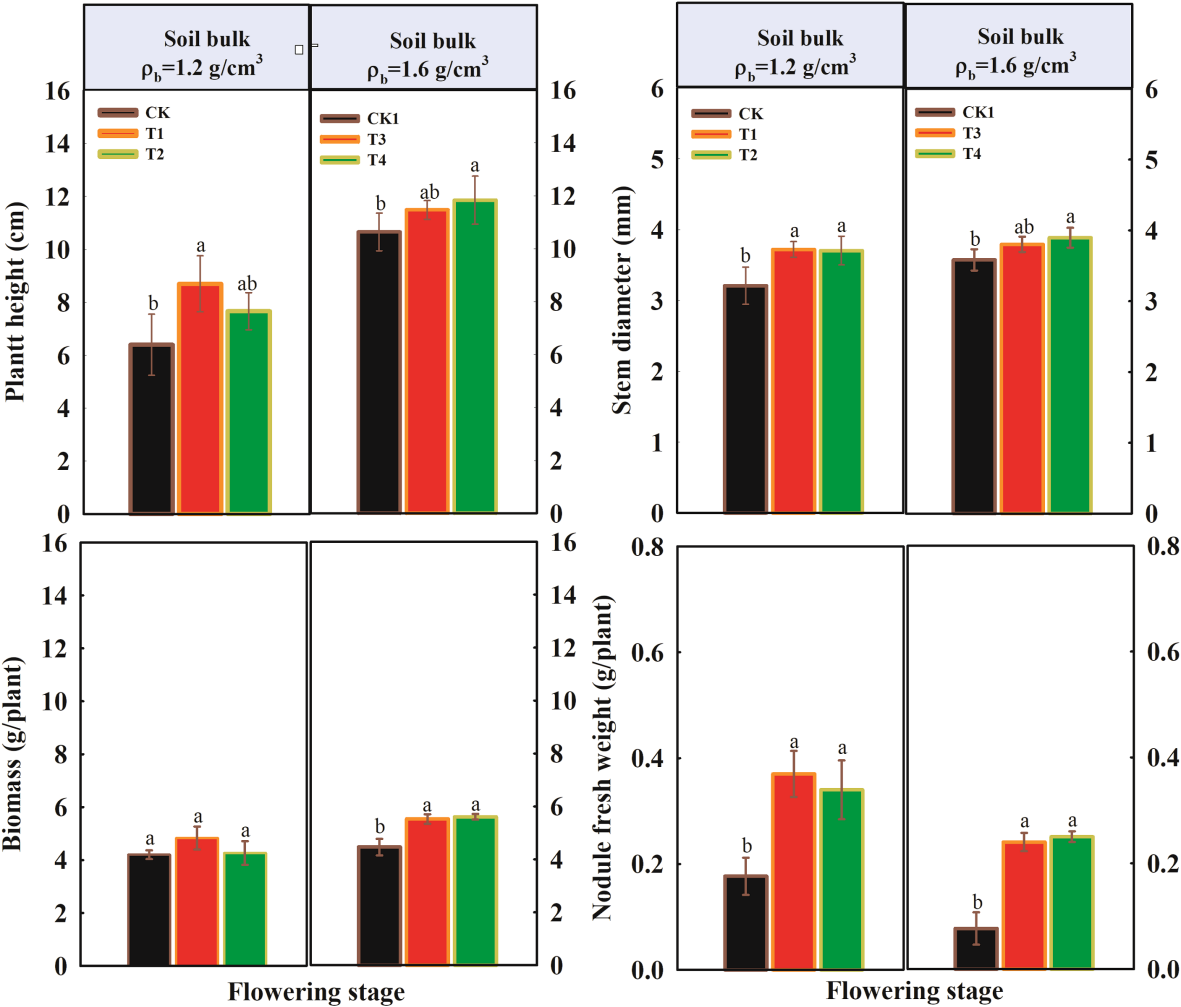
Influence of organic fertilizer under soil compaction on plant traits at flowering stage. Different letters indicate a significant difference at the *p* < 0.05.

**Figure 2:**
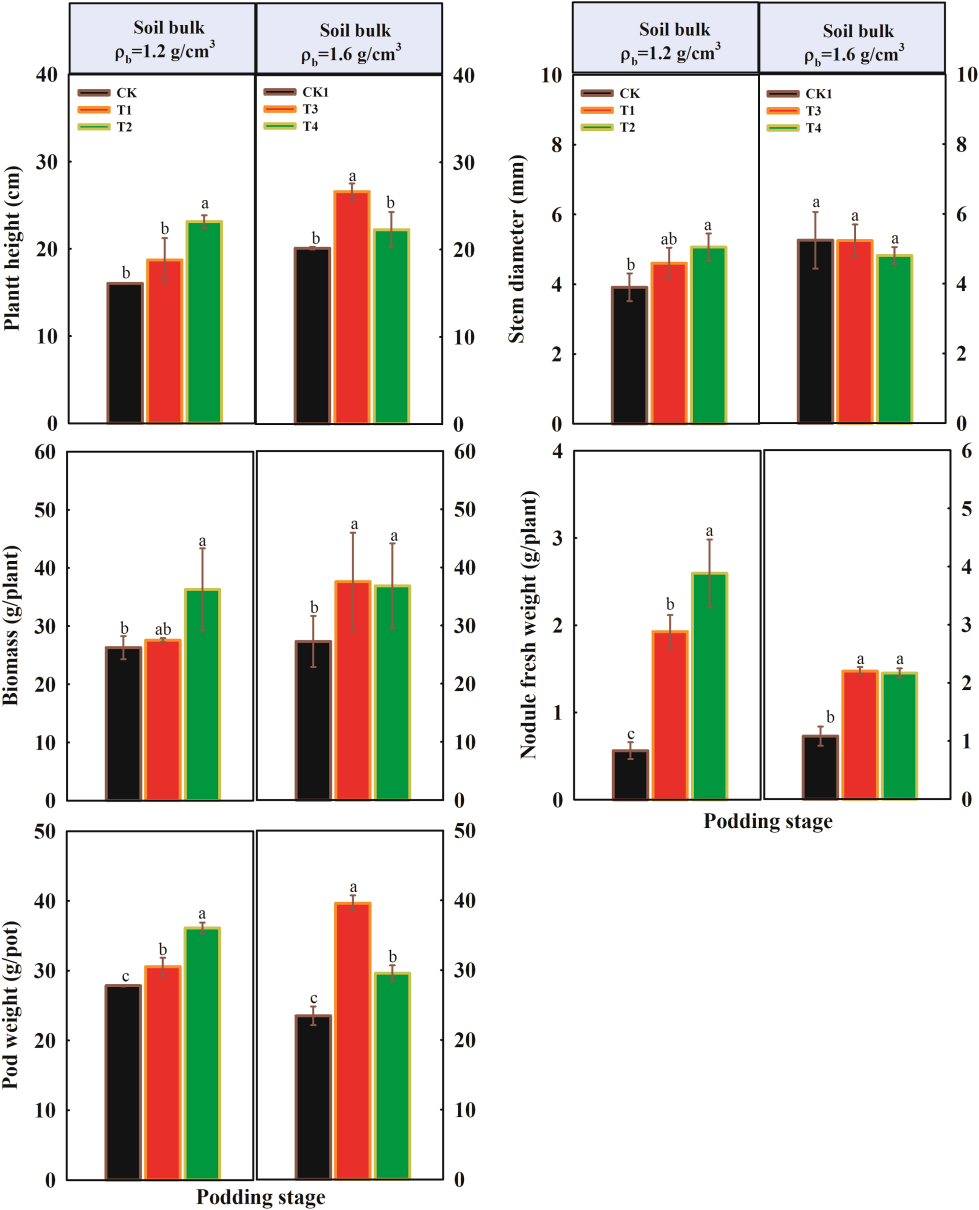
Influence of organic fertilizer under soil compaction on plant traits at podding stage. Different letters indicate a significant difference at the *p* < 0.05.

The plant traits of peanut over the growing seasons are shown in **Figure 1** and **Figure 2**. Across the two growing stages, the effects of different proportions of organic fertilizer substituting chemical fertilizer on peanut plant traits under two soil compaction levels were significant. At the flowering stage, under normal soil compaction (1.2 g/cm^3^), the highest plant height, stem diameter, and nodule fresh weight were observed in the T1 treatment, followed by T2, with the lowest values recorded in the CK (**Figure 1**). Compared to CK, plant height increased by 35.1% and 21.3% in T1 and T2, respectively. Under soil compaction stress (1.6 g/cm³), plant height, stem diameter, biomass, and fresh nodule weight increased with higher organic fertilizer application rates. For nodule fresh weight, the application of organic fertilizer had the most significant promotional effect under both soil compaction conditions.

At the podding stage, the impact of organic fertilizer on peanut growth parameters differed from that at the flowering stage (**Figure 2**). Under normal soil compaction (1.2 g/cm^3^), plant height, stem diameter, plant biomass, and nodule fresh weight all increased with higher organic fertilizer application rates compared to CK. However, under soil compaction stress (1.6 g/cm³), the highest organic fertilizer application rate (T4) led to a decrease in peanut growth parameters compared to T3.

### 3.2 The effect of organic fertilizers under soil compaction on peanut root traits

**Figure 3:**
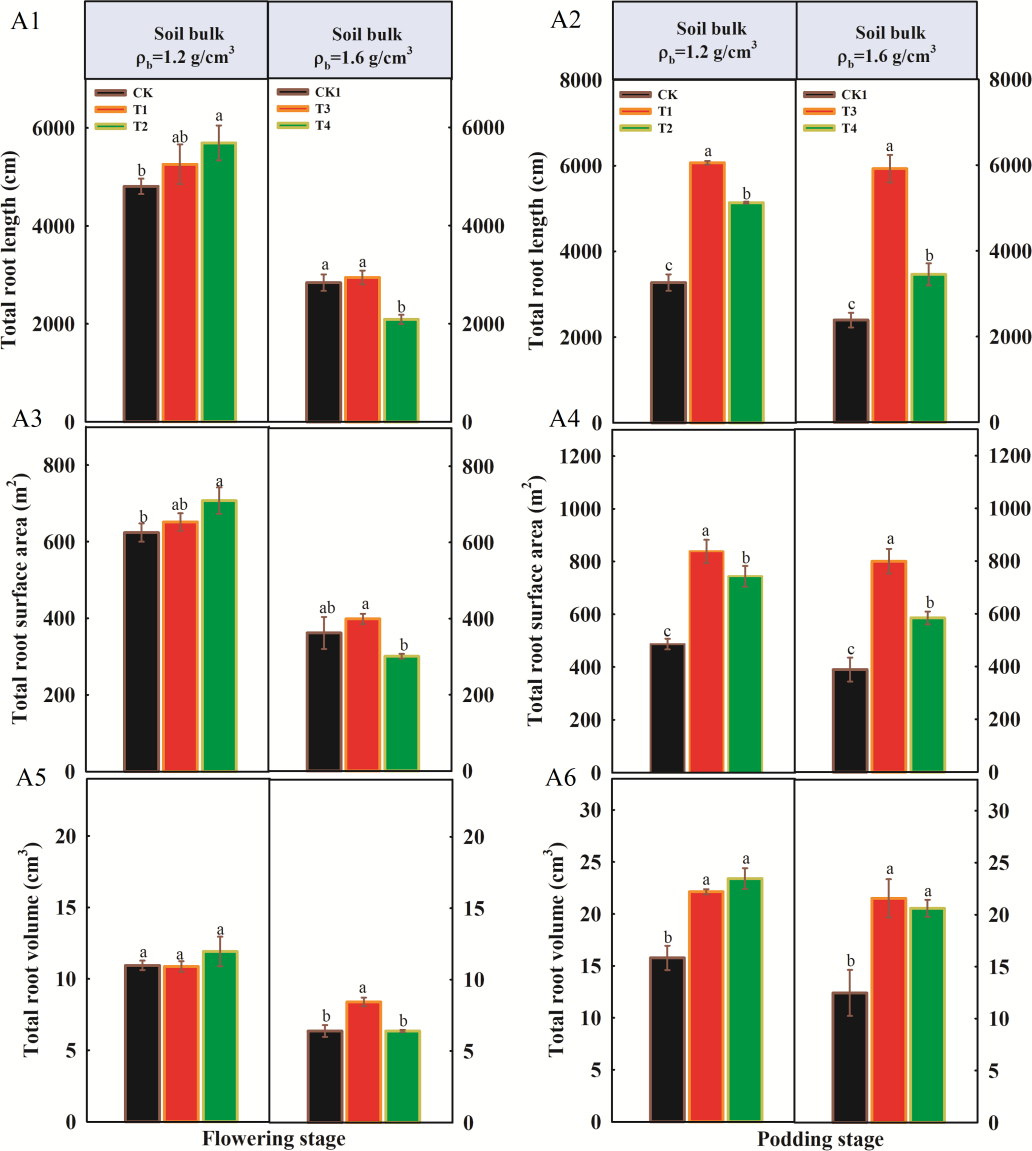
Influence of organic fertilizer under soil compaction on root traits at two growth stages. **(A1, A2)** Total root length at flowering and podding stage; **(A3, A4)** Total root surface at flowering and podding stage; **(A5, A6)** Total root volume at flowering and podding stage. Different letters indicate a significant difference at the *p* < 0.05.

The total root length, root surface area, and root volume of peanut under different soil compaction levels during two growing stages are shown in **Figure 3**. The root traits of peanut were significantly affected by the organic fertilizer application rate. At the flowering stage, compared to CK, the total root length and total surface area significantly increased with increasing fertilizer application rate under normal soil compaction (1.2 g/cm^3^), while no significant impact of different treatments on total root volume was observed (**Figures 3A1, A3, A5**). As soil compaction stress increased, the total root length, total surface area, and root volume also increased in the T3 treatment but decreased in T4. Compared to CK1, the total root length, total surface area, and root volume in T3 increased by 3.8%, 10.2%, and 31.9%, respectively; however, they decreased by 26.3%, 16.9%, and 0.2% in the T4 treatment, respectively (**Figures 3A2, A4, A6**).

**Figure 4:**
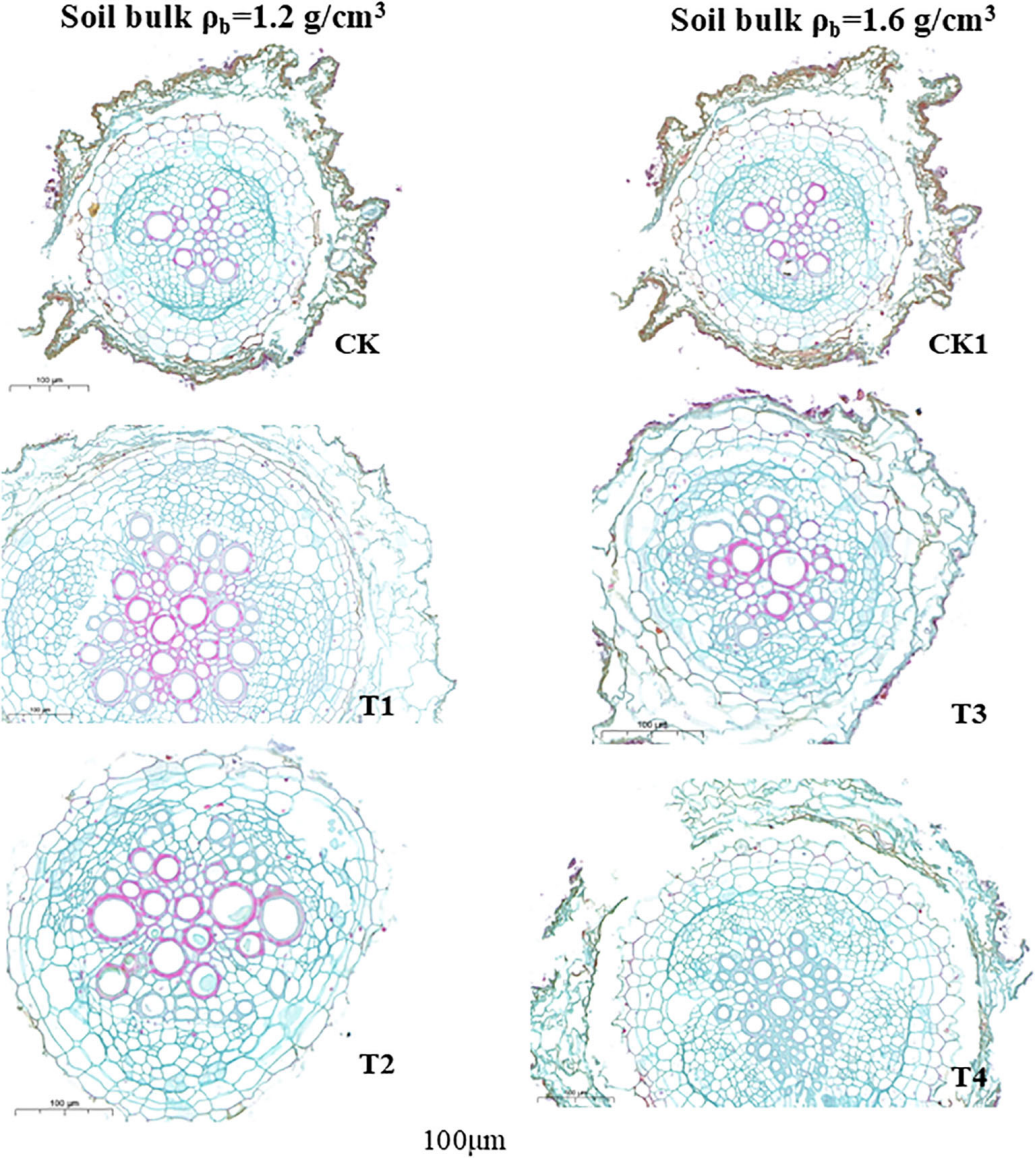
Influence of organic fertilizer under soil compaction on histochemical sections of primary root apex of peanut root. xy represents Xylem Vessel, ep represents the epidermis.

The cross-section of the primary root apex under different soil compaction levels is shown in **Figure 4**. Under 1.2 g/cm^3^ soil compaction, organic fertilizer application increased the number of xylem vessels and caused them to thicken compared to CK. In the cross-section of the primary root apex under the T2 treatment, the structure was distinct, with large epidermal cells, a wide cortical area, and a vascular cylinder in the internal part. However, under 1.6 g/cm^3^ soil compaction stress, organic fertilizer application had no obvious effect on root structure. The red lignified cells appeared lighter blue, accompanied by thinner, irregular cell shapes under severe soil compaction stress. Xylem vessels became smaller and thinner.

### 3.3 The effect of organic fertilizers under soil compaction on soil enzymes and soil nutrition

**Figure 5:**
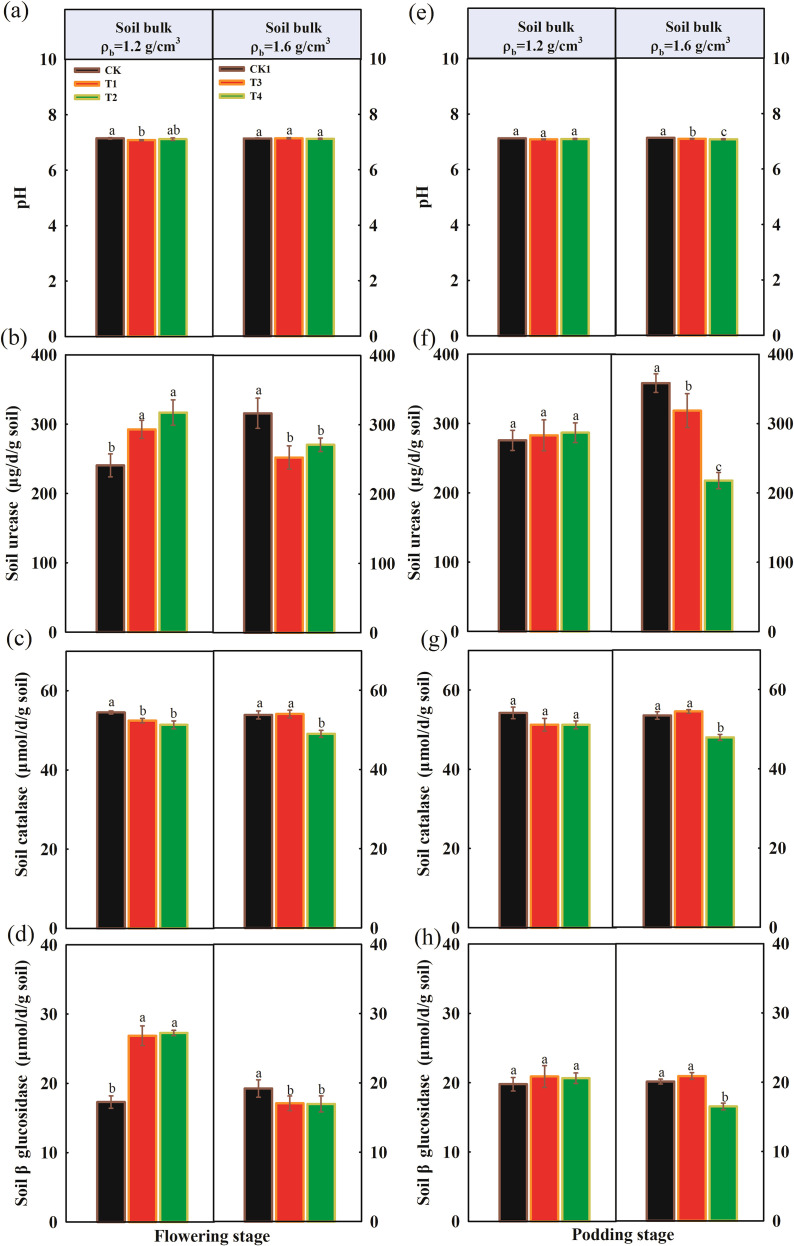
Differences in soil enzyme activities and soil βglucosidase under soil compaction level with organic fertilizer application. **(a)** pH at flowering stage; **(b)** soil urease (µg/d/g soil) at flowering stage; **(c)** soil catalase (μmol/d/g soil) at flowering stage; **(d)** soil β glucosdase (µmol/d/g soil) at flowering stage; **(e–h)** soil enzyme activities and soil β glucosidase at podding stage. Different letters indicate a significant difference at the *p* < 0.05.

The pH, soil urease, soil catalase, and soil β-glucosidase activities over two growing stages are shown in **Figure 5**. During the flowering stage, under suitable compaction (1.2 g/cm^3^), the application of organic fertilizer significantly affected soil enzyme activity. Compared with the CK, the T1 and T2 treatments significantly increased the activities of soil urease and β-glucosidase, while decreasing catalase activity and soil pH. However, under severe compaction stress (1.6 g/cm^3^), the effects of organic fertilizer application on soil enzyme activity were opposite to those observed during the flowering stage. With increasing organic fertilizer application, soil enzyme activities significantly decreased. We also analyzed available nitrogen, available phosphorus, available potassium, and cation exchange capacity of soil over the two growing stages (**Supplementary Figure 1**). During the flowering stage, under 1.2 g/cm^3^ compaction, organic fertilizer application had no significant effect on soil available nitrogen and cation exchange capacity, but significantly affected soil available phosphorus and available potassium. Compared with CK, the contents of available phosphorus and available potassium were highest under treatment T1, followed by treatment T2. Under severe compaction stress, the contents of soil available phosphorus and available potassium gradually increased with increasing organic fertilizer application. During the podding stage, under both compaction levels, organic fertilizer application had no significant effect on soil available nitrogen, but the contents of soil available phosphorus, available potassium, and cation exchange capacity were significantly higher than those of the control.

### 3.4 The effect of organic fertilizers under soil compaction on soil bacterial composition and diversity and abundance

**Figure 6:**
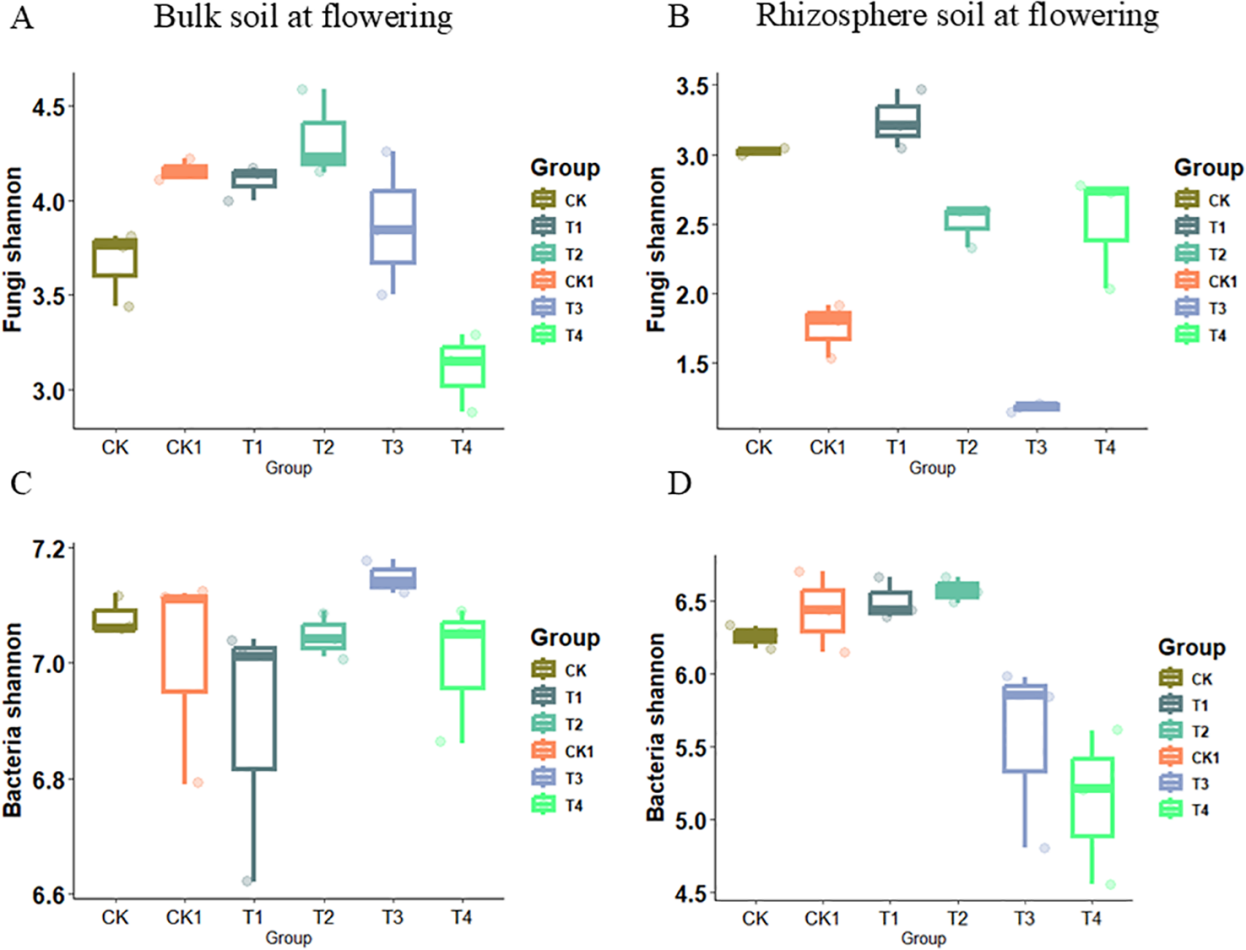
The observed bacterial and fungal Shannon indices under soil compaction level from bulk soil and rhizosphere soil at flowering stage. **(A)** Bulk soil at flowering-Fungi Shannon; **(B)** Rhizosphere soil at flowering-Fungi Shannon; **(C)** Bulk soil at flowerin-Bacterial Shannon; **(D)** Rhizosphere soil at flowering-Bacterial Shannon. ars show the maximum (top edge) and minimum (lower edge) percentiles, and boxes the 25% and 75%percentiles. The median (50%) percentile is represented by the horizontal line within the box.

**Figure 7:**
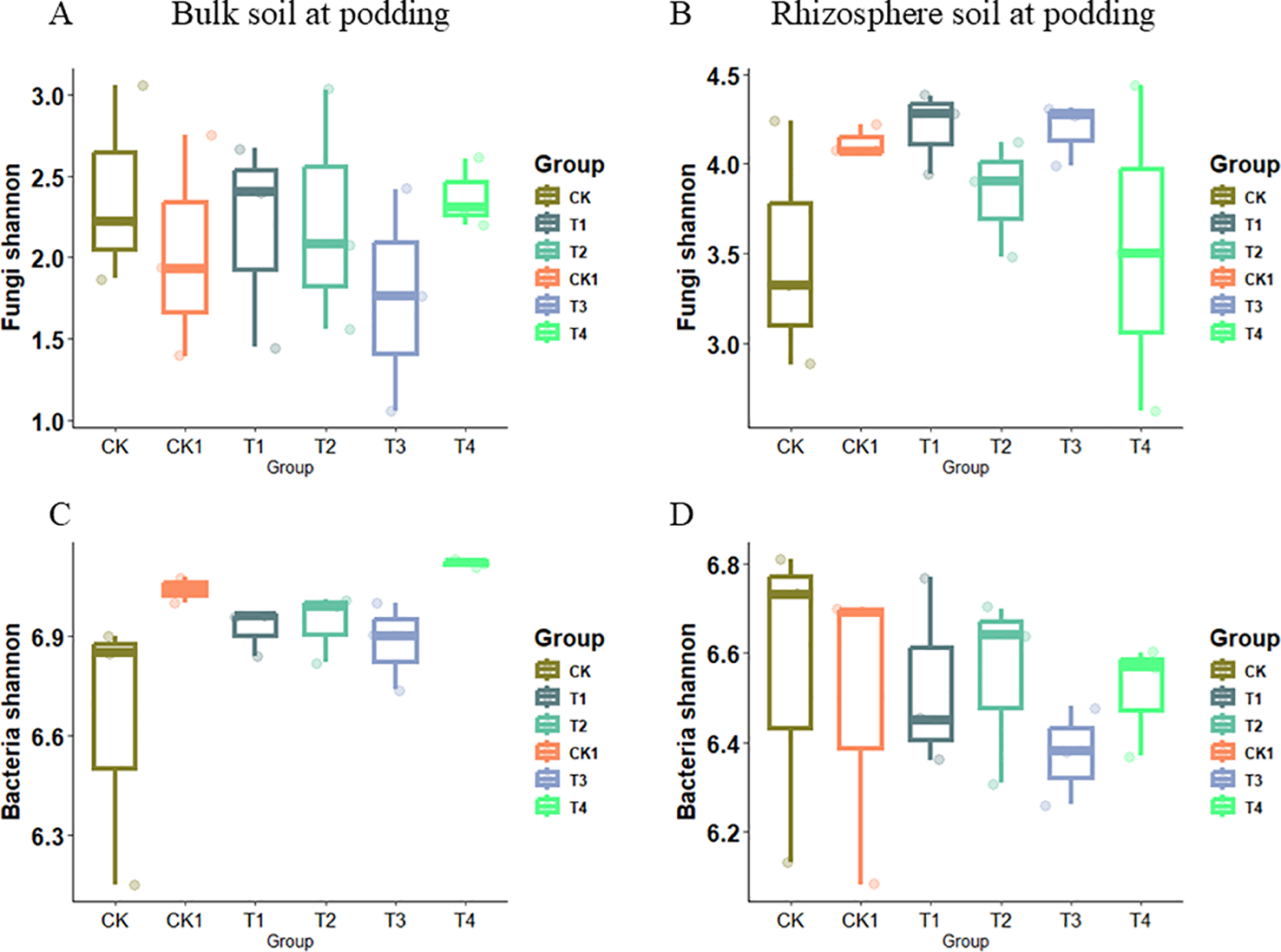
The observed bacterial and fungal Shannon indices under soil compaction level from bulk soil and rhizosphere soil at podding stage. **(A)** Bulk soil at podding-Fungi Shannon; **(B)** Rhizosphere soil at podding-Fungi Shannon; **(C)** Bulk soil at podding-Bacterial Shannon; **(D)** Rhizosphere soil at podding-Bacterial Shannon. Bars show the maximum (top edge) and minimum (lower edge) percentiles, and boxes the 25% and 75% percentiles. The median (50%) percentile is represented by the horizontal line within the box.

Microbial diversity was clearly affected by organic fertilizer application rate under soil compaction. Organic fertilizer application significantly increased fungal Shannon diversity indices. At the flowering stage, the fungal Shannon diversity index increased from 3.7 to 4.3 on average across the bulk soil compartments of CK, T1, and T2 in response to organic fertilizer. However, the fungal Shannon diversity indices decreased in the T3 and T4 treatments compared to CK1 (**Figure 6A**). In the rhizosphere soil, the fungal Shannon diversity indices showed the opposite trend, decreasing in the T2 treatment under the 1.2 g/cm^3^ compaction level (**Figure 6B**). For the bacterial community, the Shannon diversity indices in bulk soil were not significantly affected by organic fertilizer under either compaction level (**Figure 6C**). In rhizosphere soil, the bacterial community had greater Shannon diversity indices in T1 and T2, but decreased in T3 and T4 as the organic fertilizer rate increasing (**Figure 6D**). At the podding stage, organic fertilizer application did not significantly affect the Shannon diversity indices of bacterial and fungal communities under either compaction level (**Figure 7**).

**Figure 8:**
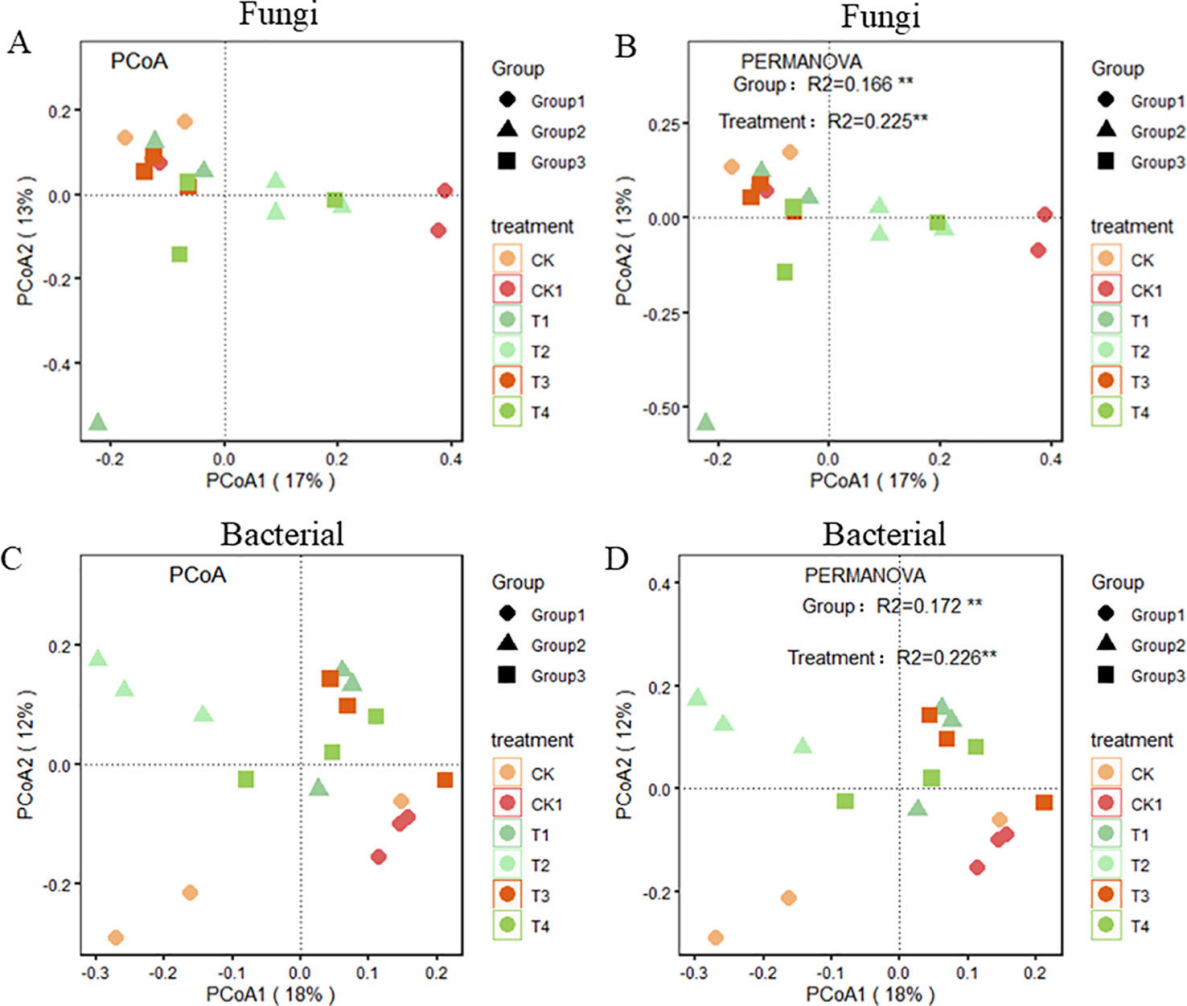
Beta diversity analysis based on organic fertilizer gradient treatments. **(A–D)** Principal coordinate analysis based on Unifrac distance matrix showing differences in Fungi and bacteria β-diversity under different soil compaction level.

In terms of β-diversity, principal coordinate analysis (PCoA) based on weighted UniFrac distance was performed on the rhizosphere fungal communities under different treatments (**Figure 8**). The PCoA with weighted UniFrac distances revealed significant differences among the organic fertilizer treatments. As shown in the figure, PCoA1 and PCoA2 accounted for 13% and 17% of the community variation, respectively. Significant differences were observed in the rhizosphere fungal communities among different organic fertilizer treatments under various compaction levels. A similar trend was observed for bacterial β-diversity.

**Figure 9:**
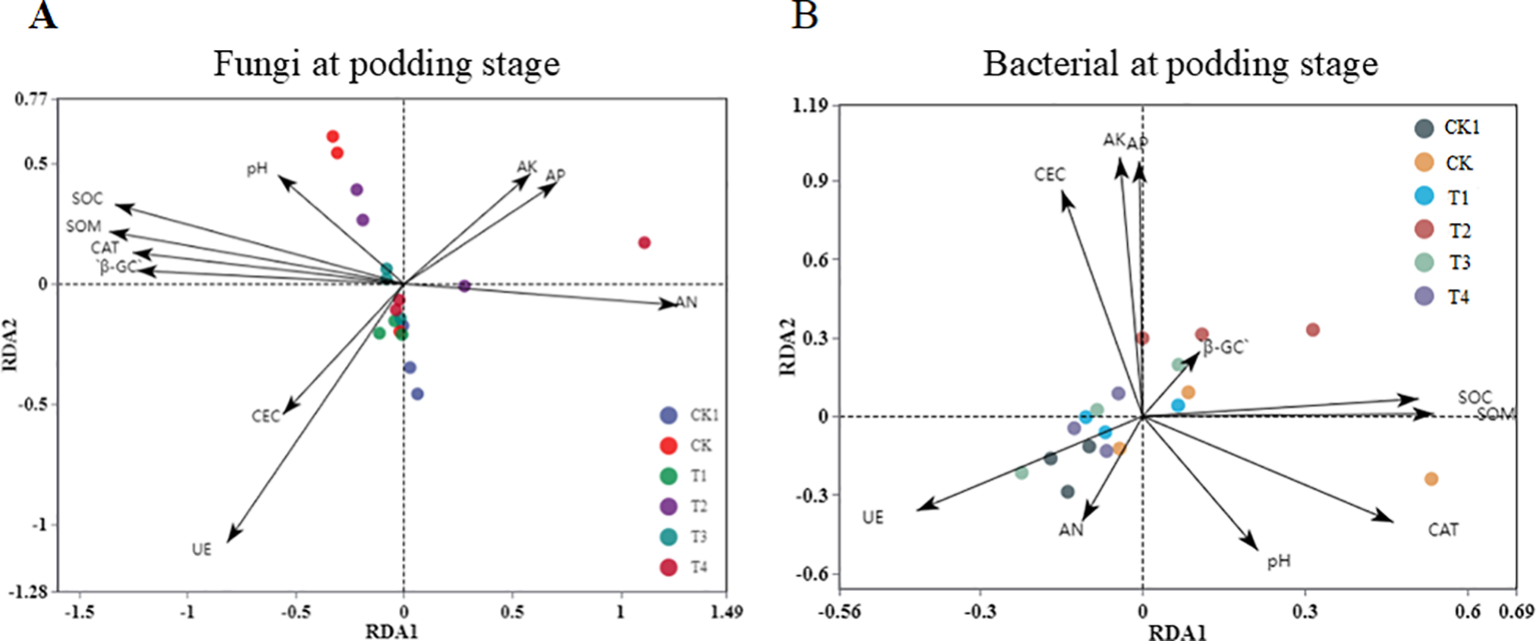
Distance-based redundancy analysis (RDA) of soil physical and chemical properties for fungi **(A)** and bacteria **(B)**. The arrows in the figure represent different environmental factors. When the angle between the arrows is acute, it indicates a positive correlation between the two factors, and when the angle between the arrows is obtuse, it indicates that the two factors are negatively related.

**Figure 10:**
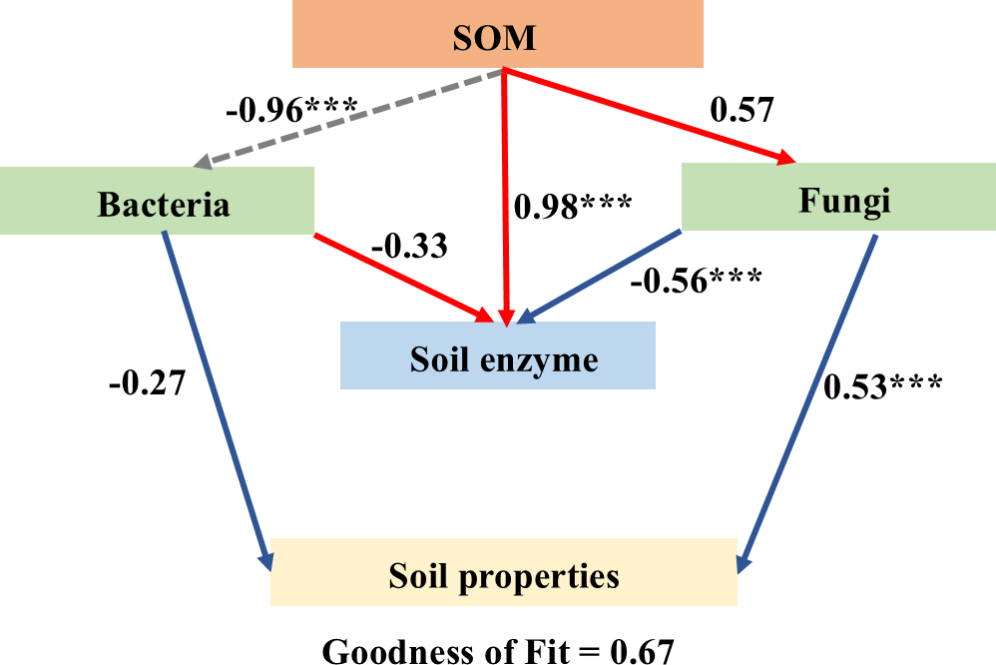
Correlation between soil properties, bacterial and fungal community as estimated using structural equation modeling (PLS-PM) under soil compaction treatments. Significance levels are indicated as: *** *p*<0.001.

To further clarify the interaction between soil physical and chemical properties and rhizosphere microorganisms, we performed a distance-based redundancy analysis (RDA) (**Figure 9**). We found that rhizosphere microbial community diversity was affected by soil physical and chemical properties. Additionally, partial least squares path modeling (PLS-PM) was used to explore the direct and indirect effects of soil physical and chemical properties and organic fertilizer application on microbial community diversity (**Figure 10**). The model showed that soil enzymes and soil properties significantly affected fungal community diversity. It was observed that soil enzymes had a direct negative influence on the fungal community with organic fertilizer application. Bacterial diversity was negatively correlated with SOM. Furthermore, SOM was positively correlated with soil enzymes, which could indirectly affect fungal diversity.

## 4 Discussion

This study found that under the condition of replacing chemical fertilizer with organic fertilizer, during the flowering stage, under moderate soil compaction (1.2 g/cm^3^), the plant height and biomass of peanut plants were optimal under the T1 treatment. Under 1.6 g/cm^3^ compaction stress, high organic fertilizer application (T2) significantly promoted plant growth and alleviated the inhibitory effects of soil compaction stress on peanut growth. During the podding stage, under moderate compaction, with increasing organic fertilizer application, plant height, biomass, fruit weight, and nodule fresh weight all increased significantly. However, under severe compaction stress, the promoting effect of high organic fertilizer application on plant growth decreased. This indicates that under compaction stress, an appropriate amount of organic fertilizer can significantly mitigate damage caused by compaction, while under severe compaction stress, even high amounts of organic fertilizer cannot fully alleviate the damage. This is primarily attributed to the optimized regulation of soil nutrient conditions and the multifaceted promotion of crop metabolism and growth following organic fertilizer application (Gu et al., 2019; Hou et al., 2025). The study found that an appropriate application of organic fertilizer significantly increased soil phosphorus and potassium content (**Supplementary Figure 1**) and soil enzyme activities (**Figure 5**) under moderate soil compaction. Soil urease, catalase, and β-glucosidase activities significantly increased, activating soil nutrients and promoting nutrient absorption by crop roots. These changes played a crucial role in crop growth.

Firstly, the increase in available phosphorus, potassium, and soil enzyme activities directly enhanced peanut nutrient uptake. The activation of soil nutrients is conducive to peanut root system growth, and our research supports this conclusion. Under moderately compacted soil conditions, organic fertilizer application significantly promoted peanut root growth, with marked increases in total root length and root surface area (Shen et al., 2016). However, under severe soil compaction stress, even a high application rate of organic fertilizer could not prevent inhibition of peanut root growth (**Figure 3**) (Yang et al., 2022). The changes in root apex cross-sectional structure also support this observation (**Figure 4**). Under high compaction conditions, the relative proportion of the vascular cylinder cross-sectional area was altered, which may affect the efficiency of axial transport of nutrients and water. Studies have shown that the response of the vascular cylinder to mechanical impedance differs between radicle and lateral roots. The vascular cylinder area increases with increasing compaction in radicles, whereas it may decrease in lateral roots (Colombi and Walter, 2017). when organic fertilizer applied, the root structure of organic fertilizer treatments (T1, T2) was superior to that of the control under 1.2 g/cm³ conditions. However, under high compaction conditions of 1.6 g/cm³, the alleviating effect of organic fertilizer was significantly weakened. Compared with CK1, the improvement in root apex anatomical structure in T3 and T4 treatments was limited. These results indicate that under severe compaction stress, soil nutrient activation is suppressed, leading to a decline in root absorption capacity and restricted growth (Liang et al., 2024).

Secondly, under soil compaction, the results showed that organic fertilizer application significantly increased the fungal Shannon diversity index in soil, consistent with previous research. For example, Bhardwaj et al. (2026) pointed out that organic fertilizer improves soil structure, providing richer habitats and nutrient sources for microorganisms, thus promoting microbial diversity. However, this study also found that under high organic fertilizer application rates (T3 and T4), the fungal Shannon diversity index decreased. This may be because excessive organic fertilizer leads to an overabundance of certain nutrients, inhibiting some fungi. Additionally, Yue et al. (2024) showed that excessive organic fertilizer application may alter the structure and function of soil microbial communities, thereby affecting microbial diversity. Bödeker et al. (2009) found that this is related to the restriction of fungal hyphal expansion due to reduced soil porosity. Fungi are key drivers of soil organic matter decomposition, and their decreased diversity directly affects the mineralization rate of recalcitrant organic compounds. RDA analysis (**Figure 9**) revealed that fungal communities were significantly positively correlated with soil organic carbon (SOC) and soil organic matter (SOM), indicating that the maintenance of fungal diversity is crucial for organic matter turnover. Under 1.2 g/cm³ conditions, organic fertilizer treatments (T1, T2) enriched lignin-degrading microbial groups within Ascomycota and Basidiomycota, facilitating the decomposition of complex organic compounds and nutrient release from organic fertilizers (Zhang et al., 2025). However, under 1.6 g/cm³ conditions, the reduced fungal diversity in T3 and T4 treatments led to decreased functional redundancy, weakening the soil’s responsive capacity to organic matter inputs, as manifested by limited enhancement of soil enzyme activities (e.g., β-glucosidase) (**Figure 9**).

For the bacterial community, this study found that under both compaction levels, organic fertilizer application did not significantly affect bacterial Shannon diversity in bulk soil. This may be because bacteria respond quickly to soil environment changes, maintaining relatively stable community structures. However, in rhizosphere soil, bacterial Shannon diversity was higher in T1 and T2 treatments, but under high compaction conditions (1.6 g/cm³), rhizosphere bacterial diversity decreased with high fertilizer application rates (T3, T4) at the flowering stage (**Figure 6**). This decrease in rhizosphere bacterial Shannon diversity may reflect changes in the structure of nitrogen-cycling functional microbial groups. Under high compaction conditions, reduced soil aeration and increased mechanical stress inhibit aerobic microbial activity while promoting anaerobic metabolic pathways (Pandey et al., 2021). We observed decreased bacterial diversity in T3 and T4 treatments, which was associated with changes in soil C/N ratio caused by high-dose organic fertilizer input and the formation of potential anaerobic microenvironments. Studies have shown that long-term excessive application of organic fertilizer can reduce the abundance of nitrogen fixation genes (nifH) while increasing the abundance of denitrification genes (nirK, nosZ) (Ma et al., 2025), leading to increased risk of nitrogen loss and decreased biological nitrogen fixation efficiency.

At the podding stage, organic fertilizer application did not significantly affect bacterial or fungal Shannon diversity under either compaction level. This may be because, at podding, plant nutrient demand increases, and microbial community structure and function are more influenced by plant growth stage than fertilizer application rate. Wang et al. (2024) also highlighted the important influence of plant growth stage on rhizosphere microbial community structure and function.

Finally, the PLS-PM structural equation model (GoF = 0.67) systematically elucidated the complex relationships among soil organic matter, microbial communities, soil enzyme activity, and soil properties. Key findings include: (1) SOM exerted an extremely strong direct positive effect on soil enzyme activity (0.98***); (2) fungal diversity served as a critical bridge linking SOM to soil property improvement (0.53***); and (3) bacterial diversity showed high sensitivity to high-dose organic fertilizer input (-0.96***), with its decrease potentially indicating impaired nitrogen cycling functions. These results provide solid quantitative support for the conclusion that “soil microbial structure was improved,” while revealing the critical regulatory role of soil physical constraints (compaction) on microbially mediated soil functions.

In summary, this study shows that the impact of organic fertilizer application rate on soil microbial diversity is jointly regulated by soil compaction and plant growth stage. Under moderate compaction, moderate organic fertilizer application increases soil microbial diversity, but excessive application may inhibit it. Additionally, soil compaction and plant growth stage have important effects on microbial community structure and function. Therefore, in agricultural production, organic fertilizer should be applied judiciously according to soil compaction degree and plant growth stage to optimize soil microbial community structure, improve soil fertility, and increase crop yield.

## 5 Conclusion

Based on the findings of this study, varying proportions of organic fertilizer used to substitute chemical fertilizer significantly affected peanut plant growth under both normal and compacted soil conditions (p < 0.05). At the flowering stage, under normal soil compaction (1.2 g/cm³), the T1 treatment achieved the highest plant height, stem diameter, and nodule fresh weight, along with increased root length and surface area. Compared to the control (CK), plant height increased by 35.1% in T1 and 21.3% in T2. Under soil compaction stress (1.6 g/cm³), plant height, stem diameter, biomass, and nodule fresh weight all increased with higher rates of organic fertilizer application. At the podding stage, under normal soil compaction, plant height, stem diameter, biomass, and nodule fresh weight also increased with higher organic fertilizer rates compared to CK. However, under soil compaction stress, excessive organic fertilizer application (T4) led to reduced growth parameters compared to T3, with total root length, surface area, and volume in T4 even declining relative to CK. Additionally, under normal compaction, organic fertilizer significantly increased soil urease and β-glucosidase activities but decreased catalase activity and soil pH compared to CK. In contrast, under compacted soil conditions, soil enzyme activities generally declined. Organic fertilizer application significantly increased fungal Shannon diversity indices in bulk soil at the flowering stage. However, bacterial Shannon diversity indices in bulk soil was not significantly affected by organic fertilizer at either compaction level. In rhizosphere soil, bacterial diversity was higher in T1 and T2 but declined in T3 and T4 with increasing organic fertilizer rates. At the podding stage, organic fertilizer had not significant effect on the Shannon diversity indices of bacterial and fungal communities under either compaction level. These results indicate that appropriate application of organic fertilizer can effectively enhance peanut growth traits, stimulate beneficial soil enzyme activities, and improve soil fungal diversity, thereby contributing to better plant performance and soil ecological health. However, under severe soil compaction, the beneficial effects of organic fertilizer are diminished. In fact, high application rates under compacted conditions can negatively impact both plant growth and soil properties, indicating that soil compaction limits the positive outcomes of organic fertilizer use.

## Data Availability

The datasets presented in this study can be found in online repositories. The names of the repository/repositories and accession number(s) can be found below: https://www.ncbi.nlm.nih.gov/, PRJNA1353603.
