# Supplementary material for: Organic fertilizer altering root trait and microbial composition to promote the compactness tolerance of peanut
Source: Front Plant Sci. 2026 Apr 22;17:1783977. doi: 10.3389/fpls.2026.1783977 (PMC13147184; doi:10.3389/fpls.2026.1783977)
Supplement: Supplementary file 1 [file SupplementaryFile1.docx]

Supplementary Material

## Supplementary Figures


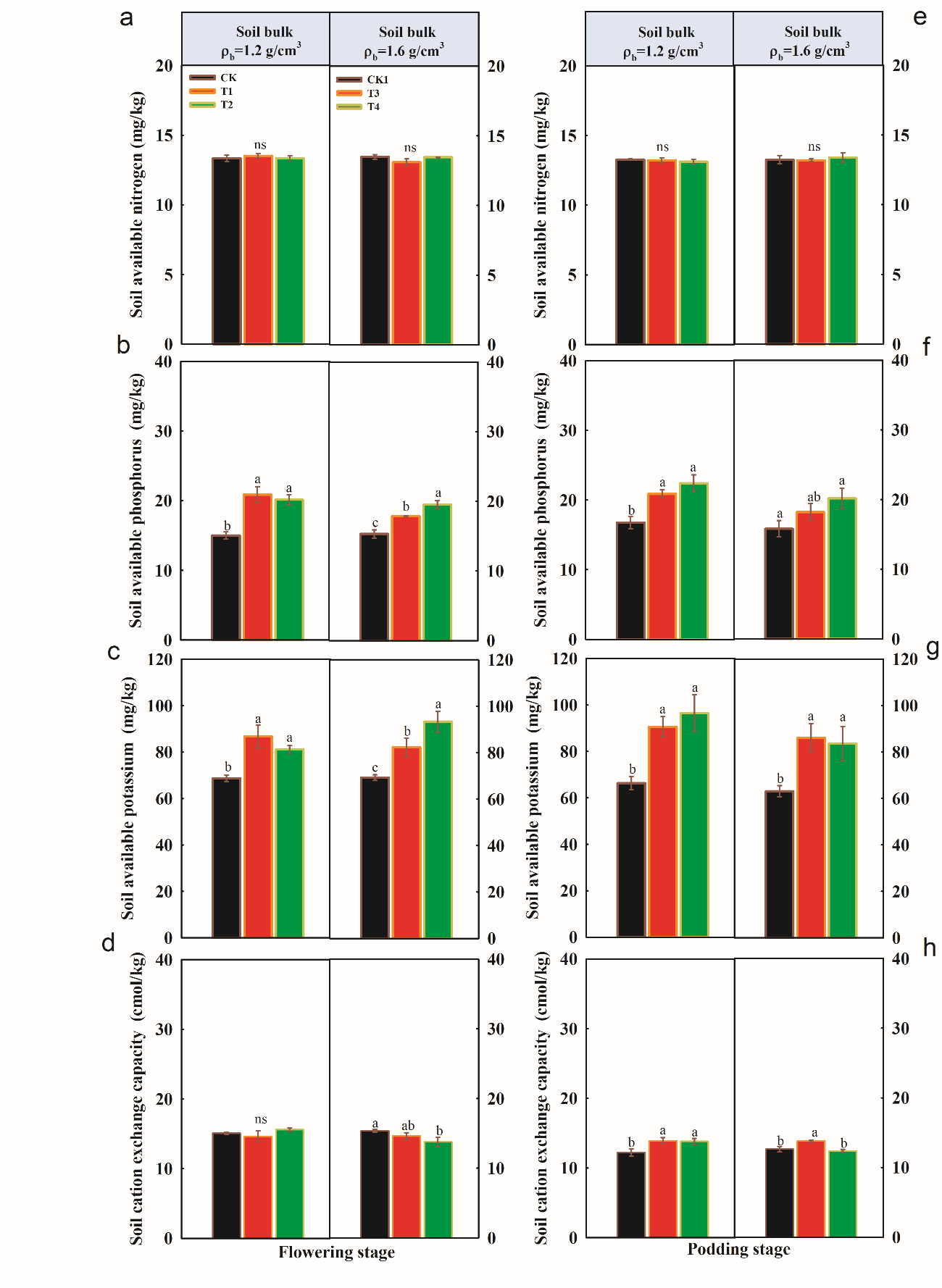


**Supplementary Figure 1.** Differences in soil nutrients under soil compaction level with organic fertilizer application. Different letters indicate a significant difference at the p < 0.05, ns, not sign.
